# Transforming liver transplant allocation with artificial intelligence and machine learning: a systematic review

**DOI:** 10.1186/s12911-025-02890-3

**Published:** 2025-02-24

**Authors:** Lisiane Pruinelli, Kiruthika Balakrishnan, Sisi Ma, Zhigang Li, Anji Wall, Jennifer C. Lai, Jesse D. Schold, Timothy Pruett, Gyorgy Simon

**Affiliations:** 1 Department of Family, Community and Health Systems Science, University of Florida, Gainesville, Florida US; 2 Department of Surgery, University of Florida, Gainesville, Florida US; 3 Institute for Health Informatics, University of Minnesota, Minneapolis, MN USA; 4 Division of General Internal Medicine, University of Minnesota, Minneapolis, Minnesota USA; 5 Department of Biostatistics, University of Florida, Gainesville, Florida USA; 6 Baylor University Medical Center in Dallas, Dallas, Texas USA; 7 Department of Medicine, University of California, San Francisco, California USA; 8 Departments of Surgery and Epidemiology, University of Colorado Anschutz Medical Campus, Aurora, Colorado USA; 9 Department of Surgery, University of Minnesota, Minneapolis, Minnesota US

**Keywords:** Artificial intelligence, Machine learning, Organ allocation, Liver transplantation, Transplant-related benefit

## Abstract

**Background:**

The principles of urgency, utility, and benefit are fundamental concepts guiding the ethical and practical decision-making process for organ allocation; however, LT allocation still follows an urgency model.

**Aim:**

To identify and analyze data elements used in Machine Learning (ML) and Artificial Intelligence (AI) methods, data sources, and their focus on urgency, utility, or benefit in LT.

**Methods:**

A comprehensive search across Ovid Medline and Scopus was conducted for studies published from 2002 to June 2023. Inclusion criteria targeted quantitative studies using ML/AI for candidates, donors, or recipients. Two reviewers assessed eligibility and extracted data, following PRISMA guidelines.

**Results:**

A total of 20 papers were included, synthesizing results into five major categories. Eight studies were led by a Spanish team, focusing on donor-recipient matching and proposing machine learning models to predict post- LT survival. Other international studies addressed organ supply-demand issues and developed predictive models to optimize LT outcomes. The studies highlight the potential of ML/AI to enhance LT allocation and outcomes. Despite advancements, limitations included the lack of robust transplant-related benefit models and improvements in urgency models compared to MELD.

**Discussion:**

This review highlighted the potential of AI and ML to enhance liver transplant allocation and outcomes. Significant advancements were noted, but limitations such as the need for better urgency models and the absence of a transplant-related benefit model remain. Most studies emphasized utility, focusing on survival outcomes. Future research should address the interpretability and generalizability of these models to improve organ allocation and post-LT survival predictions.

## Introduction

Liver transplantation (LT) is the therapeutic, life-saving intervention of choice for most patients with end-stage liver disease. The evolving landscape of healthcare has seen an increasing adoption of machine learning (ML) - including Artificial Intelligence (AI) - techniques, to enhance decision-making processes [1–5]. However, LT is a complex procedure influenced by numerous factors, including recipient and donor characteristics, organ availability, logistics, and surgical considerations, and translation of this complexity into computational solutions to improve outcomes is a challenge [2, 6]. To add to that complexity, ethical and practical aspects of organ allocation and transplantation need to be considered in policy and practice. For instance, several AI and ML techniques have been applied to organ allocation, each with its strengths and weaknesses. While these methods have demonstrated promise in various studies, no single approach has yet emerged as the clear leader in the field neither being clinically implemented.

The principles of urgency, utility, and benefit are fundamental concepts guiding the ethical and practical decision-making process for organ allocation, while aiming to avoid futility [7–9]. These principles are rooted in the ethical framework guiding public trust and integrity in organ donation and transplantation globally [10]. Various transplant stakeholders, including policymakers under the Organ Procurement and Transplant Network (OPTN) [11–13] have developed allocation policies aiming to balance these principles and ensure a fair and equitable distribution of organs. Several studies have articulated the need for advances in this field intended to move from an urgency model to a transplant-related survival benefit model [7, 14]; however, operationalizing this concept in such a way that would maximize the overall survival of all patients in need (considering both those who are waiting and those undergoing transplantation) has been a critical challenge still to be solved.

The current model in place (i.e., MELD 3.0) is primarily governed by the principle of urgency in which the sickest patients have priority for transplantation. This allocation strategy does not consider post-LT survival (principle of utility). The ideal model would use the principle of survival benefit from the starting point of an intention to transplant, i.e., it would maximize the overall survival of all patients from the point of LT listing taking into account the highest LT-related survival benefit [7, 13–17]. Although attempts have been made toward achieving such a benefit model [8, 15, 18], no model has been adopted due to important limitations, such as imprecision of survival benefit results and omitting other LT outcomes and risk factors [8, 14, 15, 18, 19]. Limitations of prior models are likely a result of limited patient-centric and longitudinal data used in previous studies; thus, preventing from capturing patient disease variability beyond MELDNa. Further, these models fail in showing how, and if, waitlist interventions have a significant impact on transplantation outcomes, and if successful, how such benefit-based model can balance these principles.

With the increasing volume of data generated in healthcare since the inception of electronic health records (EHR) [20, 21], specifically considering multi-source clinical data currently available for research, ML has emerged as a powerful tool to extract valuable insights and support personalized decision-making processes. Due to its nature of handling large amount of data and its interactions, ML models also have the potential to aid in models capable of optimizing organ allocation, predicting patient outcomes, and identifying novel risk factors while addressing important principles and clinical problems. Such resulting models would align with the ethical considerations inherent in organ allocation and transplantation decision-making, and move organ allocation and transplantation to a new scientific paradigm, i.e., a LT-related survival benefit model.

This study aimed to identify and analyze the data elements utilized in ML studies that specifically capture the complexity of candidates, organs and logistics factors impacting the principles of urgency, utility, and benefit in the context of liver transplantation. Specifically, we aimed to identify the ML methods applied, data source and features included in each model, and whether they targeted the principle of urgency, utility, or benefit. A comprehensive understanding of the current state of these studies is essential to understand existing gaps, and build the foundational knowledge needed to advance the field considering the current computational resources available toward a transplant-related survival benefit model.

## Materials and methods

### Search strategy

A comprehensive search was conducted across Ovid Medline and Scopus databases using the keywords “machine learning,” “artificial intelligence,” OR “algorithm,” OR “deep learning,” “neural network,” OR “supervised learning,” AND “transplant*,” OR “donat*,” OR “donor*,” AND “liver*. Search was performed in July 2023, for papers published from 2002 through June 2023 in any language. Due to the overall aim of this review, and that the expected search strategy would result in the inclusion of observational studies, this review was not registered under a protocol for systematic reviews.

### Eligibility criteria

Inclusion criteria encompassed quantitative studies using data from the MELD era (i.e., since the introduction of the MELD scoring system in 2002, the ‘MELD era’), peer-reviewed papers, applied a ML/AI technique, where the population of study was waitlisted liver candidates, donated liver organs, liver donors, and/or liver recipients. To be included, studies should have applied ML/AI techniques using input data up to the time of the liver transplant procedure, i.e., risk factors should have considered just up to the point when the recipient-donated organ match decision-making is made. The outcome of interest was whether models targeted urgency (i.e., predicting waitlist mortality), utility (i.e., predicting post-transplant survival), or benefit (i.e., predicting transplant-related survival benefit) for patients and/or grafts. Duplicated studies were excluded. To keep homogeneity of the included studies, papers were excluded if included population younger than 18 years old, combined transplantation other than liver and kidney, case reports, opinion papers, reviews, and reply letters. Reference lists from reviews were revised for any additional paper that could have been missed using the search strategy.

### Selection and data Collection

All titles and abstracts were screened for meeting inclusion criteria. Those papers not meeting inclusion criteria were excluded and the reason for exclusion recorded. Papers where the inclusion criteria could not be determined by reviewing title and abstract, full text were reviewed. Two independent reviewers assessed the eligibility of studies and extracted relevant data from each included paper. Any disagreement and/or not clarity on whether a paper met the criteria for inclusion was further discussed between the two reviewers.

The data collection tool included: authors’ names, year of publication, journal, country, aim (problem and proposed solution), experimental design, model architecture, methodological contribution if any, baseline models for comparison, data preprocessing, sample size, single or multi-site, type and number of variables (recipient, donor, logistic, other), input data selection, prediction target, maximization goal (urgency, utility, benefit), imputation techniques, evaluation metrics, data source (registry, EHR, other), type of data sampling (longitudinal, cross-sectional), data availability (public or not), code availability, model accuracy, high level results, and notes and/or comments.

### Risk of bias assessment

The risk of bias for each included study aimed to determine the rigor of the reported research, specifically considering ML and AI-based models, based on multidisciplinary guidelines [22] Each study was assessed for whether there was a clear reporting of the cohort building, data sources and/or settings, including inclusion and exclusion criteria. Included studies were further evaluated about clarity on the prediction problem definition, data preparation techniques, and whether included variables and missingness were reported. Reported model design and results were evaluated for clarity around input and output features, reported number of positive and negative cases, performing metrics, and models validation approaches, such as internal and/or external validation.

### Data synthesis and reporting

**Fig. 1:**
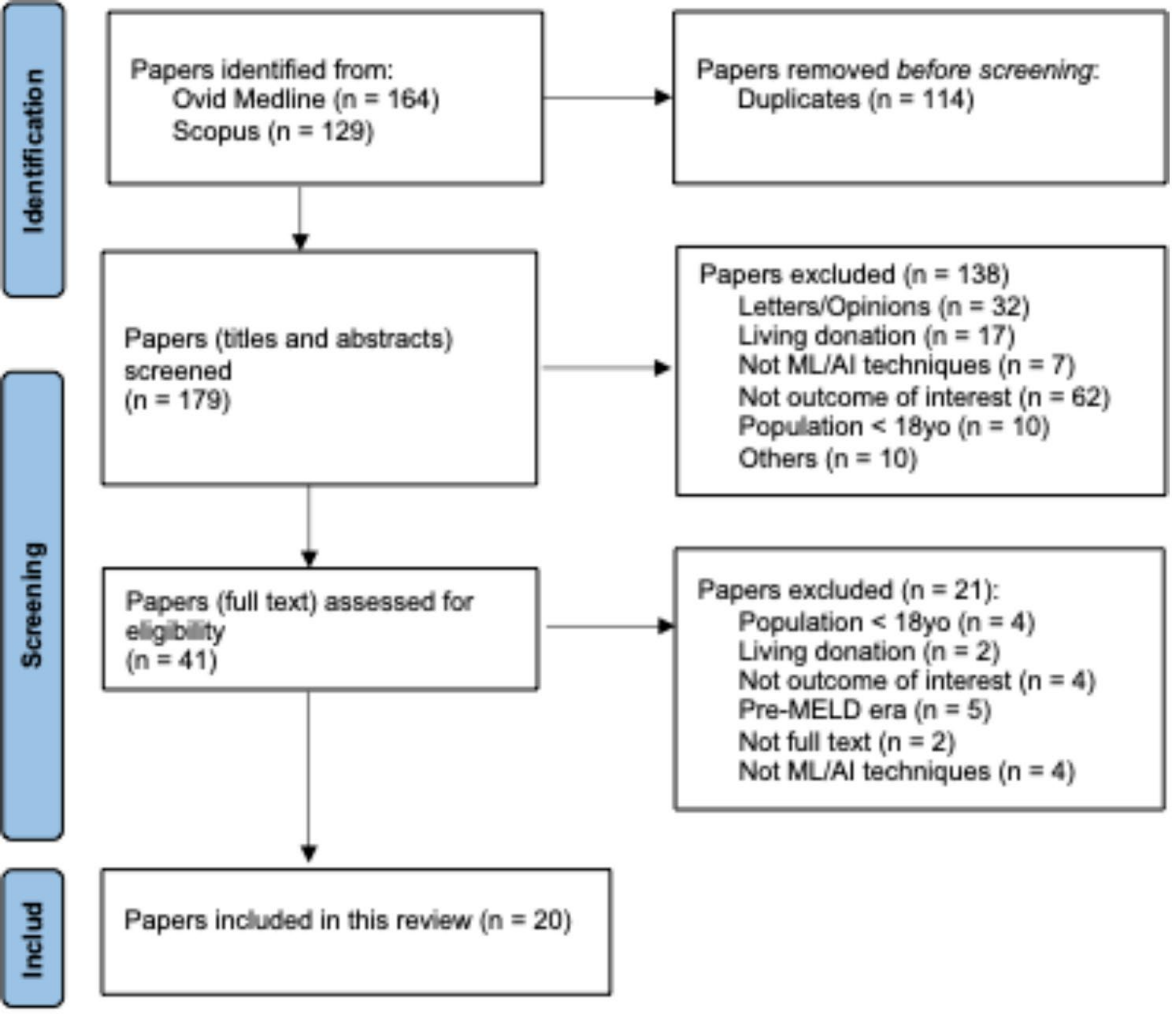
PRISMA flowchart diagram for the proposed review

The Preferred Reporting Items for Systematic Reviews and Meta-Analyses (PRISMA) guidelines [23] were followed to ensure transparency and rigor in the review process (Fig. 1). Results were grouped into five major categories based on the objectives of this review: (1) overall goal and description of the included manuscripts; (2) data sources; (3) data elements or features; (4) Principle and maximization goals; and (5) ML/AI modeling approach. Each category were evaluated based on the risk of bias before mentioned specific for that category.

## Results

A total of 20 papers were included in this review (Table 1). We present the synthesized results into five major categories. We further synthesize several papers from a single team (the Spanish team) into one major group of manuscripts, as they capture different stages of the same overall project, and synthesizing together would better capture the coverage and relevance of the project.

**Table 1:** Descriptive of the included papers (*n* = 20)

Paper	Data Source type and number(location)	Sample (*n*)	Outcome (positive outcome rate/%*)	Number of input features used	Categories of features			Best performing model	Types of models	Main performing metrics
					Recipient (*n*)	Donor (*n*)	Operative (*n*)			
Cruz-Ramírez et al. (2011) [24]	11 Hospitals (Spain)	1001	12-month graft mortality (16.1)	42	19	20	3	MPDENN_C, MPDENN_MS	Classification (Neural Network)	MPDENN-E C = 84.46; MPDENN-MS MS = 45.55
Cruz-Ramírez et al. (2011) [25]	11 Hospitals (Spain)	1003	3-month graft survival (NA)	39	16	20	3	MPDENN-E, MPDENN-MS	Classification (Neural Network)	MPDENN-E C = 89.29, MS = 13.79, RMSE = 0.3212; MPDENN_MS C = 63.89, MS = 62.07, RMSE = 0.3863
Cruz-Ramírez et al. (2012) [26]	11 Hospital (Spain)	1001	1-year graft survival (16.1)	41	16	16	9	MPDENN-E, MPENSGA-2	Classification (Neural Network)	MPDENN-E C = 83.68, MRSE = 0.3795; MPENSGA2-MS MS = 52.04, AUC = 0.5694
Cruz-Ramírez et al. (2014) [27]	11 Hospital (Spain)	1003	3-month graft mortality (NA)	57	26	19	12	NN	Classification (Neural Network)	NN-CCR (Correct classification rate) = 90.79%, NN-MS(Minimum sensitivity) = 71.42%
Briceno et al. (2013) [28]	11 Hospital (Spain)	1003	3-month graft mortality (NA)	39	16	20	3	MPENSGA-2	Classification (Neural Network)	MS = 48.98, AUC = 0.5659
Pérez-Ortiz et al. (2017) [29]	11 Hospital (UK)	822	3- and 12- month graft survival (NA)	37	16	17	4	LSVC (for 3- months), CSSVC (for 12-months)	Classification (Linear, non-Linear, Neural Network)	LSVC Acc = 90.15, CSSVC Acc = 90.15
Dorado-Moreno et al. (2017) [30]	Hospitals (7 Spain, 1 UK)	1,406	< 15-days, 15-90-days, and 90-365-days graft survival (NA)	38	16	17	5	DIM-ORNET	Classification (Neural Network)	Acc = 73.57%, geometric mean sensitivity (GMS) = 31.46%, Average mean absolute error (AMAE) = 1.155
Guijo-Rubio et al. (2021) [31]	1 Registry (UNOS, US)	39,189	3-months (7.7), 1-year (15.3), 2-years (22.1), 5-years (76.8) graft survival	28	15	11	2	LR	Classification (Linear, Decision Trees)	LR: AUC = 0.654, Acc = 0.614, MS = 0.584
Zhang et al. (2022) [32]	1 Registry (UNOS, US)	3-month: 478,777, 1-year: 47,401, 3-years: 6,380, 5-years: 45,270, 10-years: 20,751	3-month (6.4), 1-year (12.5), 2-years (21.2), 3-years (21.28), 5-years (27.8), 10-years (45.3) recipient mortality	42				XGBoost	Classification (Decision Tree)	AUC = of 0.717 for 3 months, 0.681 for 1 year, 0.662 for 3 years, 0.660 for 5 years, and 0.674 for 10 years.
Andres et al. (2018) [33]	1 Registry (SRTR, US)	2,769	0.25-year, 1-year, 3-years, 5-years, 10-years recipient survival (NA)	4	4			Cox model	Regression (Linear)	C-statistics for 0.25 year = 95.6%, 1 year = 93%, 3 year = 87.6%, 5 year = 84.1%, and 10 year = 72%
Lau et al. (2017) [34]	1 Hospital (Australia)	180	30-days (8.8), and 3-months graft failure (6.1)	15	3	12		NN	Classification (Neural Network)	AUC = 0.835
Farzindar et al. (2019) [35]	2 Registry (UNOS and SRTR, US)	87,334	Precise time of failure (Time to event) (NA)					Deep survival model	Regression (Deep survival model)	C-index = 0.82 during development and 0.57 during testing
Ershoff et al. (2020) [36]	1 Registry (UNOS, US)	57,544	90 days recipient mortality (5.4)	202	132	70		DNN	Classification (Neural Network)	AUC = 0.703
Kwong et al. (2021) [37]	1 Registry (OPTN, US)	18,920	Waitlist dropout at 3-months (6.5), 6-months (11.3), 12-months (17.2)	12	12			Cox model	Regression (Linear)	C-statistic = 0.74.
Kantidakis et al. (2020) [38]	1 Registry (UNOS, US)	62,294	Overall graft survival (NA)	97	52	45		RF and NN	Regression (Linear)	RF: C-index = 0.622 NN: IBS = 0.180
Yu et al. (2022) [39]	1 Registry (Korea)	785	1-month (8.1), 3-months (11.2), 12-months (17.2) recipient survival					RF	Classification (Decision Tree)	AUC = 0.80 for 1 month, 0.85 for 3 months, and 0.81 for 12 months
Börner et al. (2022) [40]	1 Hospital (Germany)	529	2-months, 6-months, 9-months, 12-months in-hospital recipient survival (NA)	48	24	20	4	DNN	Classification (Neural Network)	Acc = 95.8% and AUC = 0.940
Lankarani et al. (2022) [41]	1 Center (Iran)	1,947	2-years waitlist mortality (18.4)	25	25			ANN, SVM	Classification (Neural Network)	MELDNa < 23, age < 53, and ALP < 257 were the best predictors of survival in candidates
Raju et al. (2023) [42]	1 Registry (UNOS, US)	62,556	90 days recipient mortality (NA)	29	29			FT-Transformer	Classification (Neural Network)	AUC = 0.96–0.98, Acc = 0.89
Ivanics et al. (2023) [43]	3 Registry (UNOS/US, Canadian, UK)	UNOS = 59,558, Canada = 1,214, UK = 5287,	90-days recipient mortality (NA)	23	15	4	4	Ridge-Logistic regression	Classification (Linear)	AUC = 0.74 − 0.71. External model performance across countries overall had poor performed

Note: *Positive outcome rate = Number of positive outcomes (cases) divided by the total number of exposed (cases and non-cases)

### Overall goal and description of the included papers

The majority of the included papers had the overarching goal of proposing better models to address the challenge of matching donors and recipients, the current failure of successfully predicting post-LT survival at the time of the procedure, and how best to use resources available to decrease adverse outcomes considering the imbalanced low supply in front of the high demand for organs.

Out of the 20 included papers, eight of these studies were originally conducted and/or collaborated with a Spanish team [24–31]. Initially, between 2011 and 2014, internally in Spain, the team devised a model that illuminates the intricacies of donor-recipient matching in liver transplantation [24, 25, 27, 28]. The model leverages Multi-Objective Evolutionary Algorithms, diverse selection techniques, and ML models, underscoring the potential of these methodologies to enhance organ allocation systems. Starting in 2017, their work spanned from Spain to the UK [29, 30], tackling prolonged transplantation waiting times as a result of the donor shortages by introducing a ML-based donor-recipient allocation system predicting post-LT survival. Simultaneously, they criticize the prevalent reliance on MELD in the current LT allocation system, advocating for a more efficient decision-support model to enhance organ allocation [30]. Doing that, they note the absence of a scoring system capable of integrating the urgency of a transplant candidate with the optimal survival benefit among potential candidates. The authors advocate for the implementation of advanced machine learning techniques to enhance the accuracy of organ allocation predictions [31]. Also from Spain, another team [32] addresses the pressing challenges of insufficient organ donors and inadequate organ allocation. They propose that incorporating additional features and long-term predictions can reveal the impact of various risk factors on both short- and long-term outcomes post-LT.

Worldwide, teams have been investigating how to propose better models to improve organ supply and demand issues. A Canadian and Swiss team [33] proposed to identify the absence of a currently calibrated model for assessing LT outcomes, developing a calibrated model specifically designed to predict post-LT survival for Primary Sclerosing Cholangitis (PSC). In Australia, a study [34] underscores the scarcity of tools to predict graft failure or primary nonfunction at the time of LT decision-making. The same study introduces an index leveraging donor and recipient factors to predict graft failure. In the US, studies [35, 36] address the challenges posed by the shortage of organs and the scarcity of optimal donors for successful transplantation. They propose a predictive model for post-LT patient survival rates, aiming to support clinical decisions to optimize organ-recipient allocations considering the critical issue of organ demand surpassing supply, leading to patient fatalities while awaiting transplantation. The papers emphasize the importance of a predictive model for post-LT survival to prevent transplantation in cases with unacceptably low probabilities of post-LT survival.

Specifically, for Hepatocarcinoma (HCC) patients, a US study [37] addresses the persistent challenge of accurately predicting LT outcomes for this specific population by introducing a prediction model for waitlist dropout among LT candidates with HCC. Similarly, a team from the Netherlands [38] developed a model to predict post-LT survival and assert that ML holds the potential to surpass existing methods in survival prediction. A team from South Korea [39] highlights the shortcomings in existing predictive models for post-LT survival. They undertake a comparison between traditional statistical models and machine learning approaches to enhance the accuracy of predicting survival post-LT.

In Germany, a study [40] acknowledges the existence of several models for predicting post-LT survival, yet none achieve near-perfect accuracy in such a way that can provide better performance than clinical judgment. They advocate for the application of deep learning, asserting that it can yield more precise predictions for overall survival post-LT. Similar to the Spanish group, an Iranian team highlights the insufficiency of MELD as a criterion for LT allocation [41]. They propose the use of a hybrid artificial neural network (ANN) to develop a decision support system aimed at enhancing LT prioritization. A team from India [42] emphasizes the lack of a clear understanding of risk factors predicting post- LT survival. They advocate for a ML approach to establish a more effective model for predicting survival post-LT.

An international team from Canada, the US, the Netherlands, and the UK [43] recently highlighted the uncertainty surrounding the potential performance and transferability of prediction models using registry data. They utilize data from three national registries to develop ML models predicting 90-day mortality post-LT within and across countries.

### Data sources and management

The Spanish group [24–28] gathered multicentric data from 11 centers in Spain, capturing two years of longitudinal data, which accounted for the inclusion of 1003 LTs. However, the source of the data, whether it was derived from EHR or a Registry, was not clear. When collaborating with the UK [29, 30] they assembled data from King’s College Hospital in the UK, covering the period of eight years and building 858 donor-recipient pairs. They further combined with the data from the seven centers from Spain, which included 634 LT patients over two years, resulting in a dataset of 1406 donor-recipient pairs.

Among the national registries, the Scientific Registry for Transplant Recipients (SRTR) data is a common source for studies in transplantation in the US and elsewhere [31, 33, 35, 36, 38]. One specific study [43] gathered data from three different registries: the Canadian Organ Replacement Registry (CORR, Canada), the National Health Service Blood and Transplantation (NHSBT, UK), and the United Network for Organ Sharing (UNOS, US).

Sample sizes vary depending on the timeframe and the inclusion criteria, ranging mostly from a couple thousand to a hundred thousand. Single center registries and/or EHR seem to be the source for some studies; however, studies lack clarity on the different data sources and processing. With very few exceptions, there are very few or no details reporting data management and measures for data quality control across the papers. Some studies report how they transformed variables to categorical, combined others, calculated scores, but with no further details on the preprocessing steps and how inherent challenges in data management were solved. Data missingness and imputation methods were seldom reported, and when reported in a few cases, lacked clarity. However, a few papers provided further details on missingness and methods for imputation, adding to the importance of handling the inherent problem of data quality in secondary use of data and to the rigor of the proposed approach [38, 42].

Considering the data sources mainly being from registries and the methodologies employed, most of the studies used data as a cross-section; i.e., as a snapshot in a specific time point. Only one study used longitudinal data to capture risk factor change over time [27], where one of the features, MELD, was treated as a range difference between listing and operative scores. However, none of the included studies used time-dependent risk factors, associated interventions, or their progression as a source of capturing disease trajectories and/or patient deterioration while on the waitlist. Besides risk factors, target outcomes were often considered longitudinally over time; however, most of the times graft and patient survival were treated as a binary outcome at different time-windows (e.g., 1-month, 3-month), not as a time-to-event outcome.

### Data elements/features included

Most of the papers included a large number of recipient, donor, and operative (including logistics and/or from extraction to implant factors) features. Four papers included just recipients’ data. One paper [35] didn’t report how many features were included; restricting their report to stating that recipient, donor, and operative variables were included. The number of input features ranged from a couple dozen to hundreds.

Specifically for recipient features, the most often included were demographics, indication for LT, the presence and/or absence of comorbidities, dialysis, medications, MELD score, having HCC or not, and exception points conditions. Some studies included Intensive Care Unit stay at the time of LT, being on mechanical ventilation and laboratory values other than the ones for MELD score calculation. Functional status, socio-economic, and insurance factors were seldom included. With the exception of MELD that was used once as the difference between value at listing and at the time of LT, none of the input variables incorporated time-dependent variation or acuity level, and risk factors were considered as present or absent.

Donor and donated organs characteristics were mostly captured as demographics, cause of death, use of insulin and vasopressors, and the absence or presence of certain disease and comorbidities. Socio-behavioral factors such as smoking, and drugs use were included in a few studies. Serology results were often incorporated, as well as laboratories values.

Logistical features covered a broad range of information. Organ related features were often included, such as preservation solution, cold and warm ischemia time, and whole and/or split donation. Organ procurement information, i.e., donation after circulatory death (DCD) versus brain death (DBD) were seldom included [28] Considering the current advances in the organ preservation, none of the included studies considered procurement type, i.e., normothermic regional perfusion (NRP) versus super-rapid recovery (SRR), as an input feature. Some studies included compatibility between donated organ and recipient information, such as blood type, gender, and Human Leucocyte Antigen (HLA) mismatch.

Some studies incorporated previous developed scores into their models as input feature or as a comparison metric, such as the Donor Risk Index (DRI), the Survival Outcomes Following Liver Transplantation (SOFT), the Predict-SOFT (P-SOFT), Delta-MELD (D-MELD), and the Balance of Risk Score (BAR).

### Principles and maximization goals

Papers address the donor-recipient matching challenge mostly by predicting different post-LT survival outcomes. These studies address the principle of utility by using recipient and donated organ features at the time of LT to predict graft and/or recipient survival post-LT. The majority adopted a classification task, designating the outcome as a binary of graft/recipient survival or non-survival, at varying time windows post-LT. These time windows mostly include 3-months graft survival [25], 12-months graft survival [24] and 1-year graft survival [26]. Recipient survival is considered in a similar fashion, at 4-months, 1-, 3-, 5-, and 10-years post-LT [33, 36]. One recent study used the precise time of graft failure implementing a time-dependent ML technique [35], the only study that considered the outcome as a time-to-event feature. One paper considered the outcome of in-hospital recipient mortality [40].

The principle of urgency, in an attempt to develop a better model than the current MELD, aiming at maximizing waitlist survival, was the target of two papers. These studies evaluated candidate mortality within 2-years of waitlist time [41] and waitlist dropout at different time windows 3-, 6-, and 12-months from waitlist time [37]. None of the included papers provided transplant-related survival benefit models, considering a model capable of maximizing survival post-LT while minimizing mortality on the waitlist.

Among the studies included, several have demonstrated noteworthy successes, specifically in better urgency models. For example, ML was used to improve organ matching [17, 18, 20, 22], some models achieving significant performance (C-Index > 0.84) in transplant success rates. Thus, these studies demonstrated that AI-based models incorporating donor-recipient features significantly reduced organ wastage. These studies underscore the transformative potential of AI in enhancing decision-making processes in organ allocation.

### Machine learning and artificial intelligence approaches

In the included papers, several different ML techniques and some optimizations were employed to improve overall models’ performance when predicting pre- and post-LT outcomes.

Studies employed diverse data imputation techniques, feature selection methods, and validation strategies. Some studies didn’t report any imputation approach for missing values; thus, not being able to evaluate whether there were missing values or not, and if present, how they were addressed [24]. Mean imputation was a common approach in a few paper [25, 27], some with a more simplistic approach while others with a more advanced technique, such as when imputing values below 1% with mean and polynomial regression for values exceeding 1% [25]. A study [40] proposed a novel approach -Multidimensional Medical Combined Imputation (MMCI) algorithm - to address the challenge of missing values.

Feature selection methods varied among studies, with random forest being a common choice. One study [32] employed a wrapped method that integrated logistic regression with binary particle swarm optimization (BPSO) algorithm showing a better performance than other features selection methods; while achieving the same performance as if the completed dataset were used.

For validation, most of the papers adopted internal validation. Some studies employed a 75% training set and 25% testing set split [24, 27] while others reported 80 − 20% split [25] Train-test splitting strategies ranged from 67 − 27 bootstrap sampling [34] to 90 − 10 cross-validation [27]. Cross-validation was employed by using a stratified *n*-fold validation, while combining different strategies (e.g., cost-sensitive and oversampling) for imbalanced data [29, 30].

The included papers covered a limited range of ML methods, with almost the majority adopting a supervised learning. One paper employed semi-supervised learning [29] to address the problem of imbalanced datasets, as is the case of LT data, which was the concern of several studies [29, 30, 37]. The main techniques were out-of-the-shelf or modified Random Forest (RF) [32, 38, 42], and Artificial Neural Network (ANN) [24, 27, 28, 30, 39, 42]. The Spanish team introduced the Memetic-Pareto Differential Evolutionary Neural Network (MPDENN) [24, 25, 27, 28] and utilized to train various neural network models, such as generalized radial basis functions and radial Bessel function neural networks. These models demonstrated competitive performance across multiple metrics, including accuracy, root mean square error (RMSE), minimum sensitivity, and area under the curve (AUC) showing methodological contribution, specifically, for transplant research.

Most of the papers used several techniques to compare performance results. These comparison techniques included both traditional and non-traditional techniques to solve clinical problems, such as linear and multiple regression, Cox hazards, LASSO, Ridge, ElasticNet, LightGBM [28, 33, 34] The broad coverage of modeling techniques utilized across papers shows the amount of efforts teams are making in an attempt to solve the donor-recipient matching problem and provide better solutions that can add to decision-making. From the application of a single specific technique to the combination of several techniques to a specific problem, the way investigators incorporated these techniques is outstanding, from feature selection to evaluation techniques.

The included papers acknowledged various limitations. Some limitations included the retrospective nature of data collection, the need for larger and more diverse datasets, and the inherent challenges in predicting outcomes in the dynamic field of LT. The lack of a clear understanding of the risk factors for post-LT survival, the potential biases in using registry data, and the uncertainty in predicting long-term outcomes were also recognized as limitations in several papers. Another limitation is the inclusion of features commonly found in national registries; thus, lacking patient variability and other social determinants of health, specifically with the increased evidence that these factors are associated with processes of care and outcomes. The heterogeneity in data sources, patient populations, and transplant practices posed challenges in standardizing prediction models across studies.

## Discussion

This review intended to synthesize and analyze data elements utilized in ML/AI papers that specifically capture the complexity of candidates, organs and logistics factors impacting the principles of urgency, utility, and benefit in the context of LT. Despite the advancements and successes of these techniques in predicting various LT-related outcomes, and the wide array of data elements incorporated into models, several common limitations were observed, such as the lack of studies successfully developing a truly transplant-related benefit model, and very few proposing a better urgency model with potential to outperform, clinically and statistically, the current MELD score.

Most of the studies addressed the principle of utility, likely due to the high emphasis on survival as an outcome, the lack of other metrics to evaluate transplantation success, and the fact that predicting post-LT outcomes with higher accuracy at the time of LT is still a challenge to be solved. However, transplantation research has been incorporating additional metrics to examine other forms of transplant benefit, such as quality of life, life of years saved and hospital-free days post-transplantation [44–47].

Overall, the included papers provide valuable contributions into the application of ML/AI techniques for improving the prediction of LT-related outcomes, specifically looking into input features and models results; however, the interpretability and generalizability of these models remain important considerations for future research [48, 49]. Another major finding, and considering the current state of explainable AI on developing unbiased and fair models [1, 48], no study reported how their models addressed or mitigated those concerns. Additionally, external validation was seldom used, and comparison with baseline models were not consistently performed across all studies, limiting the generalizability of the results [3, 50].

Key findings varied across papers, but generally emphasized the need for advanced prediction models to enhance organ allocation, improve post-LT survival predictions, and address challenges such as organ shortages and prolonged waitlist times. ML/AI, specifically deep learning, approaches were frequently used and recommended for their potential to provide more accurate and individualized predictions. While the limitations of AI and ML in organ allocation were found significant, the successes reported in various studies indicate the transformative potential of these technologies. For example, AI has shown promise in improving the accuracy of donor-recipient matching, reducing organ wastage, and potentially extending transplant survival rates. Future work should focus on scaling these successful models, addressing current barriers, and integrating them into clinical practice.

Several papers identified common challenges in the field of LT prediction models. These challenges included the scarcity of effective prediction tools, the critical issue of organ demand surpassing supply, and the need for more precise and personalized predictions. Organ shortages, prolonged waitlist times, and the complex nature of donor-recipient matching were recurring themes across papers. However, important confounders were not included, such as transplant center effects [51, 52]. The studies underscored the limitations of traditional statistical models and the potential benefits of incorporating advanced ML techniques, specifically for its ability to handle complex interactions as the ones found in the donor-recipient matching problem [3]. The heterogeneity of data sources, the complexity of feature selection, and the uncertainty surrounding prediction model performances were also acknowledged as challenges.

This review is limited to the included papers and the studies’ results reported. As this review intended to capture large studies using ML and covered a wide time window, included papers used a retrospective approach, which is usually the case in current ML applications. However, several efforts are in place to move for more prospective studies where implementation science can be incorporated to address some of the problems raised in this review, such as the lack of transferability, external validation, and additional measures to evaluate transplantation success. It is clear that, with very few exceptions, ML in LT studies have been primarily using registry data, which lacks granularity and fails to capture longitudinal variations that are time-dependent and a few studies have shown to highly impact LT outcomes [53, 54]. The heterogeneity in data sources ranging from single centers, countries to multi-centers with different data sources, patient sub-populations, and different transplant practices poses a constant challenge in analyzing transplant data and harmonizing different datasets for prediction models.

This review poses a foundational knowledge for future studies. As many included papers pointed out, research in LT using ML faces several challenges, sometimes inherent to the data and others to the modeling approach. Despite numerous AI/ML models being applied to organ allocation, no one model has proven consistently superior across studies. While methods like Random Forests (RF) and XGBoost (XGB) have frequently demonstrated strong predictive power, their success appears to depend heavily on the dataset and specific objectives of the study. Neural networks, particularly deep learning models, have also shown promise but struggle with interpretability and require large amounts of high-quality data. Overall, it is clear that the field is still in a stage of exploration, and further research is needed to establish which techniques will prove most effective for long-term success in clinical settings. While some models show more promise than others, a consensus on the best-performing model has yet to emerge, indicating that more work is required to identify the most reliable and scalable approaches for organ allocation.

New strategies should consider the development and validation of more sophisticated ML models, the exploration of novel features and data sources, and the integration of additional clinical information to improve prediction accuracy, specifically targeting the inclusion of individual variability into models. Addressing the challenges of organ shortages and prolonged waitlist times through advanced allocation systems is also still to be solved, and although ML can provide better allocation systems, it will not solve the shortage of organs directly. It may be that a better allocation system would be able to provide enough evidence to expand donor criteria and show that some recipients would have LT-related survival benefit even if organs not currently considered transplantable are transplanted in the future. Moreover, it may be able to identify the optimal procurement technique for DCD donors and the optimal storage strategy (i.e., cold storage versus machine perfusion) for all donors that will optimize utilization of specific organs or donor types [55–57]. This could drive allocation to specify what procurement technique or storage strategy should be used for each donor to optimize utilization potential.

The need for collaborative efforts across centers, standardized and automatized data collection and harmonization, and external validation of prediction models across different regions and populations are key important considerations for future research in the field. We acknowledge that the application of ML models to organ allocation and post-transplant outcomes carries ethical considerations, particularly around the potential for algorithmic bias. To address this, various techniques to ensure fairness, such as fairness metrics and model interpretability tools, should be incorporated. These safeguards help prevent discrimination based on factors like age, sex, and geographical location, and allow clinicians to make more transparent and informed decisions. However, we also recognize that further work is needed to ensure that ML models are continuously monitored for bias and that their use in clinical practice is aligned with ethical standards.

Further, considering the current advances in donor procurement and organ preservation, new strategies to move from data silos to large and integrated multiple data sources capturing the several stakeholders and factors impacting the donor-recipient matching problem are still needed. It may be that in the new future, multiple efforts can be made to combine longitudinal recipient data (e.g., EHR), donor-organ-procurement characteristics, transplant and patient-centered socio-geographical characteristics, and novel metrics that could better capture transplantation success from a system and patient perspective; thus, providing the premises for the development and implementation of a successful transplant-related benefit model.

### Solutions and future directions

This review identifies significant limitations in the current use of ML/AI for liver transplantation, particularly the lack of granularity in registry data and the challenges in improving upon existing models. The current MELD score primarily prioritizes patients based on the severity of their liver disease (urgency). This approach does not adequately consider post-transplant survival (utility) or the overall survival benefit (benefit) derived from transplantation, neither both concepts together. To develop a benefit model that maximizes the overall survival of all patients in need, several steps can be taken, such as incorporating advanced ML techniques, such as reinforcement learning and generative adversarial networks, as well as causal AI could not just enhance model performance by better capturing the complexity of liver transplantation processes, but develop causal model where the causal effect of transplantation could be estimated. Additionally, leveraging explainable AI methods could improve the transparency and interpretability of these models, making them more acceptable to clinicians.

Moreover, integrating more granular data sources, including patient-generated health data, longitudinal clinical data derived directly from electronic health records, real-time monitoring, and multi-omics data, could provide a more comprehensive view of patient health and disease progression. This integration would allow for more accurate and personalized predictions. This review also support to establishing collaborative frameworks to combine data from multiple centers, thus increasing the robustness and generalizability of the models.

#### Limitations of this review

We acknowledge that this review may have missed important papers due to its search strategy, as not-ML papers addressing LT-related survival benefit. This review didn’t include data from patients younger than 18 to prevent bias, and doing so, the review may have missed important ML methodological contributions in the field, if pediatric LT was considered.

## Conclusions

Overall, the review provides a comprehensive review of recent research efforts in the development and improvement of LT ML models, shedding light on the data elements/features used as input data, modeling techniques, key findings, challenges, and potential future directions. The integration of advanced ML techniques and the emphasis on personalized and precise predictions, where models can capture the individual variability of patients over time, underscore the evolving landscape of LT research, and the need to develop better models that are patient-centric and account for better metrics of transplantation success in addition to survival. These findings of our review point towards several avenues for innovation that could substantially shift current practices in liver transplantation. By proposing the use of advanced machine learning techniques and integrating more granular data sources, we aim to enhance the precision and personalization of liver transplantation decisions.

## Data Availability

All data generated or analyzed during this study are included in this published article and is publicly available.

## Abbreviations

Acc, C: Accuracy
AI: Artificial intelligence
ANN: Artificial neural network
AUC: Area under the curve
BAR: Balance of risk score
C-index: Concordance index
COOR: Canadian organ replacement registry
CSSVC: Cost-sensitive support vector classifier
DBD: Donor after brain death
DCD: Donor after circulatory death
DIM-ORNET: Dynamic imbalanced ordinal neural network
DMELD: Delta-MELD
DNN: Deep neural network
DRI: Donor risk index
EHR: Electronic health records
FT-Transformer: Feature tokenizer transformer
HCC: Hepatocarcinoma
IBS: Integrated brier score
LT: Liver transplantation
LR: Logistic regression
LSVC: Linear support vector classifier
MELD: Model for end stage disease
ML: Machine learning
MPDENN-E: Memetic pareto differential evolutionary neural network- entropy
MPDENN-MS: Memetic pareto differential evolutionary neural network-minimum sensitivity
MPENSGA2: Memetic pareto evolutionary non-dominated sorting genetic algorithm-2
MS: Minimum sensitivity
NHSBT: National health service blood and transplantation
NN-Neural: Network
NRP: Normothermic regional perfusion
OPTN: Organ procurement and transplantation network
P-SOFT: Predict- survival outcomes following liver transplantation
PSC: Primary sclerosing cholangitis
RF: Random forest
RMSE: Root means square error
SOFT: Survival outcomes following liver transplantation
SRR: Superrapid recovery
SRTR: Scientific registry of transplant recipients
SVM: Support vector machine
UNOS: United network for organ sharing
XGBoost: Extreme gradient boosting
